# METHODS - A randomised controlled trial of METhotrexate to treat Hand Osteoarthritis with Synovitis: study protocol for a randomised controlled trial

**DOI:** 10.1186/s12891-021-04842-0

**Published:** 2021-11-15

**Authors:** Yuanyuan Wang, Andrew J. Teichtahl, Graeme Jones, Helen I. Keen, Catherine L. Hill, Anita E. Wluka, Jessica Kasza, Flavia M. Cicuttini

**Affiliations:** 1 Department of Epidemiology and Preventive Medicine, School of Public Health and Preventive Medicine, Monash University, 553 St Kilda Road, Melbourne, VIC 3004 Australia; 2 Menzies Institute for Medical Research, University of Tasmania, Hobart, TAS 7000 Australia; 3 Rheumatology Group, School of Medicine, University of Western Australia, Perth, WA 6009 Australia; 4 Department of Rheumatology, Fiona Stanley Hospital, Murdoch, WA 6150 Australia; 5 The Queen Elizabeth Hospital, University of Adelaide, Woodville, SA 5011 Australia; 6 Department of Medicine, University of Adelaide, Adelaide, SA 5000 Australia

**Keywords:** Methotrexate, Osteoarthritis, Hand, Pain, Function

## Abstract

**Background:**

Hand osteoarthritis is a common and disabling problem without effective therapies. Accumulating evidence suggests the role of local inflammation in causing pain and structural progression in hand osteoarthritis, and hand osteoarthritis with synovitis is a commonly encountered clinical phenotype. Methotrexate is a well-established, low-cost, and effective treatment for inflammatory arthritis with a well-described safety profile. The aim of this multicentre, randomised, double-blind, placebo-controlled trial is to determine whether methotrexate reduces pain over 6 months in patients with hand osteoarthritis and synovitis.

**Methods:**

Ninety-six participants with hand osteoarthritis and synovitis will be recruited through the Osteoarthritis Clinical Trial Network (Melbourne, Hobart, Adelaide, and Perth), and randomly allocated in a 1:1 ratio to receive either methotrexate 20 mg or identical placebo once weekly for 6 months. The primary outcome is pain reduction (assessed by 100 mm visual analogue scale) at 6 months. The secondary outcomes include changes in physical function and quality of life assessed using Functional Index for Hand Osteoarthritis, Australian Canadian Osteoarthritis Hand Index, Health Assessment Questionnaire, Michigan Hand Outcomes Questionnaire, Short-Form-36, tender and swollen joint count, and grip strength, and structural progression assessed using progression of synovitis and bone marrow lesions from magnetic resonance imaging and radiographic progression at 6 months. Adverse events will be recorded. The primary analysis will be by intention to treat, including all participants in their randomised groups.

**Discussion:**

This study will provide high-quality evidence to address whether methotrexate has an effect on reducing pain over 6 months in patients with hand osteoarthritis and synovitis, with major clinical and public health importance. While a positive trial will inform international clinical practice guidelines for the management of hand osteoarthritis, a negative trial would be highly topical and change current trends in clinical practice.

**Trial registration:**

Australian New Zealand Clinical Trials Registry (ANZCTR), ACTRN12617000877381. Registered 15 June 2017, https://www.anzctr.org.au/Trial/Registration/TrialReview.aspx?id=373124

## Background

Osteoarthritis (OA) is the most common chronic joint disease and frequently involves the hand [1]. Hand OA is a common and disabling problem worldwide, resulting in significant disease burden and impaired quality of life. Most middle-aged people have radiographic OA affecting at least one hand joint, with 20% having disabling pain [2]. The age-standardised prevalence of hand OA was 44.2% for females and 37.7% for males in the general population [3], much higher than the prevalence of rheumatoid arthritis (~1%). Hand OA is as disabling as rheumatoid arthritis, impeding activities of daily living such as dressing and eating [4], and has similar clinical importance with regard to health-related quality of life as rheumatoid arthritis [5]. Since the prevalence of hand OA increases with age, the ageing population is expected to drive increasing disease burden and healthcare costs for treating this condition [6]. Despite the high prevalence and disease burden, there is a lack of effective therapies for hand OA. Clinical guidelines for the management of hand OA, such as those from the European League Against Rheumatism suggest topical therapy (preferred over systemic treatments, topical non-steroidal anti-inflammatory drugs (NSAIDs) being first-line choice), oral analgesics (e.g. paracetamol and NSAIDs), and non-pharmacological therapy (education, assistive devices, exercises and orthoses) [7]. However, these therapies only have limited efficacy in reducing pain.

Accumulating evidence suggests that hand OA is a heterogeneous disease involving all joint structures, in which local inflammation plays a central role in causing pain and structural progression [8]. A common phenotype of hand OA is the inflammatory phenotype, characterised by joint swelling (synovitis) [9, 10], and this phenotype represents an aggressive form of disease [11–14]. Imaging studies have shown a high prevalence (approximately 50%) of synovitis in symptomatic hand OA [15, 16], with painful joints significantly more likely to have synovitis than non-painful joints [15, 17, 18]. Synovitis is the strongest predictor for radiographic progression of hand OA over 2 years [11, 12] and 5 years [13, 14], supporting inflammation is a risk factor for rapid disease progression. Therefore, inflammation is a potential treatment target in hand OA, and therapies targeting synovitis may offer a novel approach for reducing disease burden from hand OA.

Recent efforts to examine the effect of anti-inflammatory therapies in hand OA have been limited by including all patients with hand OA, not just those with the synovitis phenotype. A randomised controlled trial showed adalimumab (a tumour necrosis factor blocker) was not superior to placebo to alleviate pain in patients with hand OA not responding to analgesics and NSAIDs [19]. This study was limited by the short duration (6 weeks) and no specific phenotype (i.e. with synovitis) of hand OA being examined. There is proof of concept that targeting synovitis will be effective. In a 1-year randomised controlled trial of patients with symptomatic erosive inflammatory hand OA, etanercept (a tumour necrosis factor blocker) resulted in greater pain reduction, more radiographic remodeling, and less bone marrow lesions compared to placebo in those with inflammation [20]. In another 12-month randomised controlled trial of people with erosive hand OA, adalimumab significantly halted the progression of joint damage in those with synovitis [21]. Based on the histology of the synovium in hand OA [22], there is no biological rationale why specific therapies targeting one inflammatory pathway, such as tumour necrosis factor antagonists, provide effective treatments for hand OA with synovitis. Less specific therapies may offer greater disease modification, since multiple pre-inflammatory cytokines are targeted. Methotrexate is a well-established, low-cost, and effective treatment for inflammatory arthritis with a well-described safety profile. In a Cochrane review of 7 randomised controlled trials comparing methotrexate with placebo in rheumatoid arthritis, methotrexate monotherapy showed a clinically important and statistically significant improvement in symptoms at 52 weeks and significant reduction in radiographic progression [23]. Methotrexate reduced pain in a randomised controlled trial over 6 months and an open-label trial over 24 weeks of knee OA [24, 25]. However, a recently published randomised controlled trial showed no superiority of 10 mg methotrexate weekly over placebo in pain relief at 3 or 12 months in patients with symptomatic erosive hand OA refractory to usual treatments [26]. This study may be limited by the very low dose of methotrexate (10 mg weekly) and not inflammatory phenotype of hand OA being examined. Based on the available data, it is clinically important to determine whether targeting those with hand OA and synovitis is likely to improve the effectiveness of methotrexate on reducing pain and/or slowing structural progression of hand OA.

## Hypothesis and objectives

We propose a randomised, double-blind, placebo-controlled trial to determine the effect of 20 mg methotrexate weekly, compared to placebo, on reducing clinical symptoms and structural progression in people with hand OA and synovitis over 6 months. It was hypothesised that methotrexate will (1) reduce pain (primary hypothesis), and (2) improve physical function and quality of life (secondary hypothesis), and reduce structural progression (secondary hypothesis) over 6 months compared to placebo in people with symptomatic hand OA and synovitis. If methotrexate is proven to be effective, it will offer a novel therapeutic approach to reducing pain and disease progression of hand OA.

## Study design

The METHODS (A randomised controlled trial of METhotrexate to treat Hand Osteoarthritis with Synovitis) study is a multicentre, randomised, double-blind, placebo-controlled trial over 6 months.

## Trial registration

The trial was registered at the Australian New Zealand Clinical Trials Registry prior to recruitment commencing (ACTRN12617000877381, registered 15 June 2017; amended 22 July 2021). The trial reporting will be guided by the Consolidated Standards of Reporting Trials (CONSORT) Statement [27]. Due to the COVID-19 pandemic, the trial was temporarily halted in March 2020, when 80 participants had been randomised with 60 participants taking study medication for at least 6 months. Due to potential safety concerns at that time, participants were asked to stop study medication. The trial was resumed in November 2020. The 7 months when participants were off treatment due to safety concerns regarding the possibility of COVID infection in those taking methotrexate, would interfere with the validity of the use of the primary outcomes assessed at 2 years for hand pain and radiographic progression. However, the use of pain measured at 6 months will provide a valid measure to test whether methotrexate improves pain. In November 2020, the protocol was amended to make pain reduction at 6 months as the primary outcome, and radiographic progression at 6 months as a secondary outcome. Accordingly, the other secondary outcomes were also amended, which included changes in physical function, quality of life, joint activity, and grip strength at 6 months, and progression of synovitis and bone marrow lesion at 6 months after the amendment. The amendment was submitted to the Australian New Zealand Clinical Trials Registry in November 2020 and updated in July 2021.

## Ethics approval

Ethics approval has been obtained from Alfred Hospital Ethics Committee (290/17), Monash University Human Research Ethics Committee (10725), University of Tasmania Human Research Ethics Committee (H0016794), Central Adelaide Local Health Network Human Research Ethics Committee (HREC/18/CALHN/458), and South Metropolitan Health Service Human Research Ethics Committee (RGS0000000573). Written informed consent will be obtained from all the participants.

## Methods

### Study setting and participants

Eligible participants with hand OA and synovitis will be recruited through the Osteoarthritis Clinical Trial Network (Melbourne, Hobart, Adelaide, and Perth, Australia), from the community via advertisements and from medical practitioners.

#### Inclusion criteria

(1) aged 40-75 years; (2) a pain score of at least 40 on a 100 mm visual analogue scale (VAS) and radiological OA (Kellgren and Lawrence grade >2) in >1 joint, as recommended by the Osteoarthritis Research Society International (OARSI) clinical trials guidelines for hand OA [28]; (3) evidence of synovitis determined from magnetic resonance imaging (MRI) according to the Outcome Measures in Rheumatology (OMERACT) hand OA recommendations and with a grade ≥1 in ≥1 joint [29].

#### Exclusion criteria

(1) concomitant rheumatic disease, inflammatory joint disease, psoriatic arthritis, ankylosing spondylitis, or gout; (2) contraindication to methotrexate [e.g. renal, liver or haematological condition, cancer including skin cancer, serious infections requiring hospitalisation in the last 5 years, known or past infection with human immunodeficiency virus, hepatitis B or C, tuberculosis, or known lung disease with scarring (any fibrosis or evidence of past tuberculosis exposure on chest x-ray), concurrent regular prednisolone use, taking regular trimethoprim or bactrim antibiotics, egg or flu vaccination allergy]; (3) contraindication to MRI (e.g. implanted pacemaker, metal sutures, presence of shrapnel or iron filings in the eye, or claustrophobia); (4) unable to complete informed consent; (5) women who are pregnant, breast-feeding or trying to become pregnant, or men who father a child.

### Study timeline

**Fig. 1:**
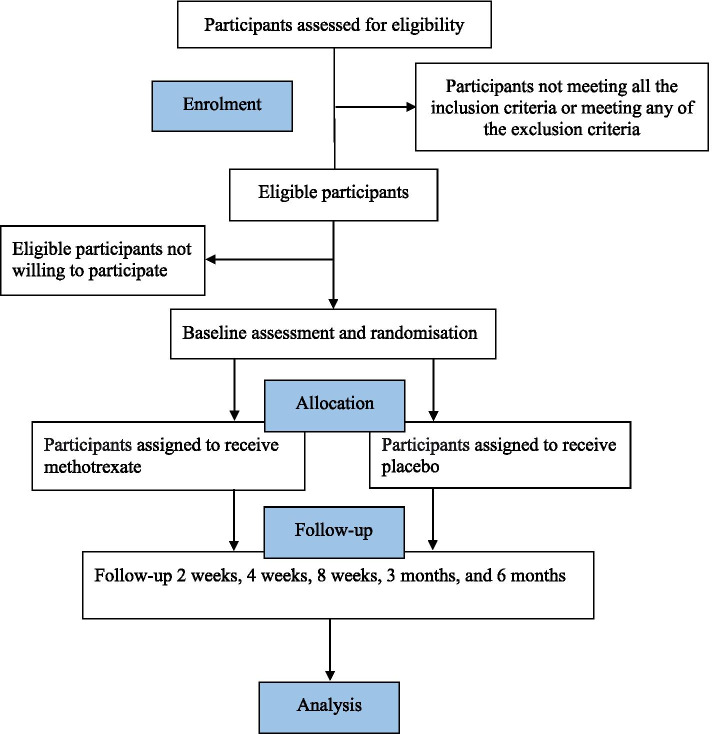
Flowchart of trial participation

This trial began recruitment in August 2017. It is estimated to accomplish recruitment in November 2021 and complete the 6-month follow-up and data collection in May 2022. Figure 1 shows trial participation and study procedure.

### Randomisation, allocation concealment, and blinding

Allocation of participants in a 1:1 ratio to one of the two groups will be based on computer generated random numbers prepared by a statistician with no involvement in the trial. Block randomisation will be performed, stratified by study site and gender, given that hand OA disproportionately influences a larger number of females than males. The use of a central automated allocation procedure with security in place will ensure the allocation cannot be accessed or influenced by any person. Participants, assessors and statisticians will be blinded to group allocation. Allocation concealment and double blinding will be ensured by: (1) medications being dispensed by hospital clinical trial pharmacy at each site; (2) use of an identical placebo tablet; (3) subjective measures being taken by research assistants blinded to group allocation; (4) objective measures of structural progression being made by trained observers blinded to group allocation and time sequence of imaging. Emergency unblinding will be allowed in limited situations that impact on the safety of study participants. Code-break for the full randomisation schedule will be maintained by the administering institute. Tamper-proof unblinding envelopes, provided by the drug company preparing the study medication, will be used in case of the need for emergency unblinding. Participants who are unblinded will be withdrawn from treatment and be requested to complete questionnaires and have a follow-up hand x-ray and MRI scan at 6 months. Given the possible toxicities related to methotrexate, there is potential for investigators reviewing blood test results to become aware of treatment. Therefore, patient-reported outcomes will be assessed before any changes in dose or results of monitoring blood test are discussed with participants. The investigators with knowledge of blood test results will not be responsible for administering questionnaires.

### Intervention

Participants will be randomised to receive methotrexate (10 mg tablets) or placebo. Placebo will be manufactured to have same colour, taste and smell as methotrexate tablets.

#### Dosing

Methotrexate is currently used in rheumatoid arthritis, commonly dosed at 20 mg per week. Given that synovitis predates rapidly progressive radiographic hand OA [11, 12], and to alleviate concerns around inefficacy due to inadequate dosing, we will follow current guidelines for use of methotrexate in rheumatoid arthritis. The maximum dose will be 20 mg per week [30]. Participants will be prescribed 10 mg methotrexate or placebo weekly for four weeks, followed by 20 mg weekly for the remainder of the study if there is no toxicity as determined at the physician’s discretion (Australian Rheumatology Association Medication Information Sheets). The decision for dose escalation will occur at the 4-week physician review. If intolerances occur (e.g. nausea), the decision to increase to 20 mg weekly can be delayed until further clinical review. In a Cochrane systematic review of methotrexate monotherapy, 16% of people discontinued methotrexate within 52 weeks due to an adverse event, compared with 8% of placebo subjects [23]. All participants will be prescribed oral folic acid at a minimum dose of 5 mg per week, to be taken on days other than when methotrexate is taken. Folic acid supplementation has been shown to ameliorate side effects of methotrexate, as well as a significant reduction in discontinuation of methotrexate [31].

#### Safety

An independent data and safety monitoring board will be convened, consisting of two independent clinical rheumatologists, a clinical pharmacologist experienced in methotrexate use, and a biostatistician, all with clinical trial experience. They will monitor adverse events and will meet bimonthly and provide a written report to the chief investigators. Adverse events will be recorded throughout the study and chief investigators will be notified of any serious events within 24 hours. Safety of treatment will be assessed according to monitoring guidelines. As recommended by the Australian Rheumatology Association, full blood count, liver function test and urea and electrolytes tests will be performed at screening, 2, 4, and 8 weeks, 3 and 6 months (Australian Rheumatology Association, Patient Information: Methotrexate). If the results remain abnormal despite action (such as reducing the methotrexate dose), or at clinician discretion, then study medication will be stopped. Further reviews with a rheumatologist will be performed at 4 weeks, 3 and 6 months.

#### Compliance

Pill counts will be performed at each clinical assessment to document compliance. Weekly telephone contact for the first 3 months will be conducted to address any concerns, as well as regular clinical reviews with a study rheumatologist (at baseline, 4 weeks, 3 and 6 months). This will help to mitigate non-compliance.

#### Concomitant medication

To maintain the pragmatic nature of the trial, there are no restrictions with regard to concomitant analgesic medications. The participants will be allowed to continue taking the treatments that they are taking at their screening visit for the duration of the trial. If a participant is experiencing pain and requires an increase in the dose of analgesics, then the use of paracetamol, topical or oral NSAIDs or opioids, or a combination of these will be permitted, but the reason for the dose increase and the dose used will be documented. Trimethoprim, which interacts with methotrexate, will not be permitted.

### Study procedure

Table 1 shows the study procedure. Volunteers will be telephone screened before undergoing in sequential order (i) clinical assessment with a rheumatologist, chest x-ray and blood tests [full blood count, kidney function test, liver function test, C-reactive protein, erythrocyte sedimentation rate, rheumatoid factor, anti-cyclic citrullinated peptide antibodies, human immunodeficiency virus, Hepatitis B (sAb, sAg and cAb), Hepatitis C, and Quantiferon Gold] to assess the eligibility of the participants and ensure no contraindication to methotrexate; (ii) x-ray of both hands (standardized posteroanterior view) to confirm radiological disease; and (iii) MRI of the study hand to determine the presence of synovitis. Consent was obtained by study doctor. The symptomatic hand will be the study hand. In case of bilateral symptomatic hands, the most symptomatic hand will be the study hand. In the case of bilateral and equal symptoms, the dominant hand will be studied. Participants are able to withdraw at any time during the trial; the time and reasons will be recorded. If participants withdraw from the study, they will be requested to complete questionnaires (posted to the participants with return envelope) and have a follow-up hand x-ray and MRI scan at 6 months or end of study where possible.

**Table 1:** Timetable and measures to be made

	Screening		Double blind period					
	Screening / baseline assessment	Post-screening	MRI & randomisation	Week 2	Week 4	Week 8	Months 3	Months 6
**Visit/phone contact**	**0**	**1**	**2**	**3**	**4**	**5**	**6**	**7**
**Informed consent**^**1**^	X							
**Blood tests**^**2**^		X						
**Safety blood tests**^**3**^				*X*	X	*X*	X	X
**Telephone follow-up**				*X*		*X*		
**Clinical visit**	X				X		X	X
**Hand x-ray**		X^a^						X^a^
**Chest x-ray**^**4**^		X						
**Hand MRI**			X					X
**Medical history including vaccination**	X						X	X
**Medications and allergies**^**1**^	X				X		X	X
**Employment and education**	X							
**Smoking**	X							
**Alcohol consumption**	X			*X*	X	*X*	X	X
**Marital status and parity**	X							
**Questionnaires**								
Hand VAS	X				X		X	X
AUSCAN, FIHOA, HAQ, MHQ	X						X	X
SF-36	X							X
Knee WOMAC, painDETECT	X							X
**Physical examination**								
Height, weight, waist and hip circumference	X							
Grip strength	X						X	X
Tender/swollen joint count^1^	X				X		X	X
Cardiovascular (including blood pressure), respiratory, abdominal, skin examination	X							
**Compliance and safety (AEs)**^**1**^				*X*	X	*X*	X	X
**Dispense medication**			X				X	
**Pill count**					X		X	X

*MRI* magnetic resonance imaging, *VAS* Visual Analogue Scale, *AUSCAN* Australian Canadian Osteoarthritis Hand Index, *FIHOA* Functional Index for Hand Osteoarthritis, *HAQ* Health Assessment Questionnaire, *MHQ* Michigan Hand Outcomes Questionnaire, *WOMAC* Western Ontario and McMaster Universities Osteoarthritis Index, *AE* adverse event

^a^Baseline and follow-up x-ray performed at the same centre using a standardized protocol

^1^To be performed/reviewed by study doctor

^2^Full blood count, kidney function test, liver function test, C-reactive protein, erythrocyte sedimentation rate, rheumatoid factor, anti-cyclic citrullinated peptide antibodies, human immunodeficiency virus, Hepatitis B (sAb, sAg and cAb), Hepatitis C, and Quantiferon Gold

^3^Full blood count, kidney function test, and liver function test

^4^Not required if chest x-ray in last 12 months is available

### Primary outcome

#### Pain reduction at 6 months

Pain reduction at 6 months will be measured according to the recommendations of the OARSI clinical trials for symptom modification in hand OA [28]. This taskforce recommended the use of a single question pain VAS as the main outcome measure for pain in hand OA clinical trials. In a recent systematic review, VAS was most frequently used for pain assessment in hand OA, with excellent reliability, good construct validity and sensitivity to change [32].

### Secondary outcomes

#### Change in pain and function at 6 months

At baseline and 6 months, hand pain, physical function, joint activity, and hand strength will be measured according to the OARSI recommendations of clinical trials for symptom modification in hand OA [28] using the Functional Index for Hand OA (FIHOA) [33], Australian Canadian Osteoarthritis Hand Index (AUSCAN) [34], Health Assessment Questionnaire (HAQ) [35], Michigan Hand Outcomes Questionnaire (MHQ) [36], Short-Form-36 (SF-36) [37], tender and swollen joint count [38], and grip strength.

#### Structural progression at 6 months assessed from MRI

Participants will undergo hand MRI for screening and a repeat MRI 6 months later using a 3.0 T or 1.5 T MRI unit according to local safety rules and standardised sequences across the 4 study sites. The participant will be positioned prone with their arm above their head with a positioning frame. Details of the sequences and parameters are shown in Table 2. Scan time is estimated at 20 minutes. Cartilage and bone will be assessed from coronal T1-weighted and proton density-weighted images. Synovitis and tenosynovitis will be assessed from coronal and axial proton density-weighted images. MRI at baseline and 6 months will be assessed using the OMERACT hand OA score (grades 0-3) for synovitis and bone marrow lesions [29]. Readings will be performed by trained observers blinded to clinical information, time sequence of MRIs, and group allocation. Progression of synovitis or bone marrow lesions will be defined as an increase in grade ≥ 1 from baseline to follow-up.

**Table 2:** Magnetic resonance imaging sequences and parameters

	Machine	T1-weighted fat-saturated 3D gradient-echo acquisition, coronal	Proton density-weighted fat-saturated acquisition, coronal	Proton density-weighted fat-saturated acquisition, axial
**Melbourne**	3.0-T whole body MR unit (SIEMENS, Skyra)	repetition time 700 msec; echo time 12 msec; flip angle 120 degrees; field of view 24 cm; 512×352 matrix; pixel 0.43*0.43; slice thickness 0.5 mm	repetition time 2400 msec; echo time 65 msec; flip angle 150 degrees; field of view 24 cm; 640×440 matrix; pixel 0.375*0.375; slice thickness 2 mm	repetition time 4870 msec; echo time 50 msec; flip angle 150 degrees; field of view 14 cm; 160×320 matrix; pixel 0.41*0.41; slice thickness 2 mm
**Hobart**	1.5T whole-body MR unit (GE, Optima)	repetition time 700 msec; echo time 20 msec; flip angle 90 degrees; field of view 22 cm; 512×512 matrix; pixel 0.43*0.43; slice thickness 0.6 mm	repetition time 2400 msec; echo time 60 msec; flip angle 160 degrees; field of view 24 cm; 1024×1024 matrix; pixel 0.23*0.23; slice thickness 2 mm	repetition time 4870 msec; echo time 50 msec; flip angle 160 degrees; field of view 13 cm; 512×512 matrix; pixel 0.25*0.25; slice thickness 2 mm
**Adelaide**	3.0-T whole body MR unit (SIEMENS, Skyra)	repetition time 700 msec; echo time 12 msec; flip angle 120 degrees; field of view 22 cm; 512×408 matrix; pixel 0.43*0.43; slice thickness 0.5 mm	repetition time 2400 msec; echo time 54 msec; flip angle 150 degrees; field of view 22 cm; 640×520 matrix; pixel 0.34*0.34; slice thickness 2 mm	repetition time 4870 msec; echo time 42 msec; flip angle 150 degrees; field of view 18 cm; 512×416 matrix; pixel 0.35*0.35; slice thickness 2 mm
**Perth**	1.5-T whole body MR unit (SIEMENS, Aera)	repetition time 550 msec; echo time 11 msec; flip angle 120 degrees; field of view 24 cm; 512×360 matrix; pixel 0.47*0.47; slice thickness 0.5 mm	repetition time 2420 msec; echo time 60 msec; flip angle 150 degrees; field of view 24 cm; 640×520 matrix; pixel 0.375*0.375; slice thickness 2 mm	repetition time 4870 msec; echo time 48 msec; flip angle 150 degrees; field of view 14 cm; 220×320 matrix; pixel 0.44*0.44; slice thickness 2 mm

#### Radiographic progression at 6 months

Radiographs will be performed at baseline and 6 months with a standardised posteroanterior view with the beam focused on the head of the third metacarpal, using a hand map for reproducibility of position, as recommended by OARSI clinical trials for imaging in hand OA taskforce [39]. One reader will score radiographs according to the OARSI atlas for progression of hand OA [40], blind to clinical information, time sequence of x-rays, and group allocation. Our reproducibility is high (kappa 0.7 to 0.9) [41]. Progression will be defined by any increase in Kellgren and Lawrence grade in any joint as previously described [3] and recommended by the OARSI taskforce for outcomes in clinical trials of hand OA [39], for joints with synovitis and for both hands. This method is endorsed by a systematic review as the most valid, reliable and sensitive to change [42].

### Other measures

#### Descriptive data

Age, gender, height, weight, duration of symptoms, employment, medical history, medication use, marital status, education level, smoking, alcohol consumption, parity and menopausal status.

#### Adverse events, analgesic use, and co-interventions

These will be measured in a log-book and by structured questioning by the blinded assessor at each follow-up.

#### Biochemical parameters

General (cell counts, liver and renal function), inflammatory markers (C-reactive protein, erythrocyte sedimentation rate).

### Sample size calculation

The average VAS pain in a previous clinical trial of symptomatic hand OA similar to the proposed study was 54 mm (standard deviation 20 mm) [43]. The minimal clinically important difference to be detected in OA trials is a 15 mm change in VAS pain (out of 100 mm) [44]. We will require 38 participants in each group to attain a power of 90% to detect the minimal clinically important difference (alpha 0.05, two-sided significance). Accounting for an estimated 20% loss to follow-up, we will recruit 96 participants, 48 in each group.

### Statistical analyses

Analyses will be conducted in Stata v16 or later. An intention-to-treat analysis, including all participants in their randomised groups regardless of their adherence to assigned treatments, will be performed in a blinded fashion. The effect of intervention on pain reduction and other pain and function outcomes measured at multiple time points (for pain: week 4, month 3 and month 6; for secondary pain and function measures, month 3 and month 6) will be analysed using mixed linear regression models, with a random intercept for participant. Models will be adjusted for the baseline measure and the stratifying variables of site and gender, and include categorical terms for time, a term for randomised group and terms for the interaction between randomised group and time. Results will be presented as mean differences between groups at each time point with 95% confidence intervals, and p-values will also be reported. For measures only taken at baseline and a single follow-up time point, regression models will omit random intercepts and terms for time. Regression modelling assumptions will be assessed graphically. In the case of a gross departure from normality of residuals, ranked based techniques will be applied. As stipulated by OARSI clinical trial recommendations [28], binary logistic regression will be used to assess the effect of intervention on structural progression at 6 months, adjusted for the stratifying variables of site and gender. The outcome of tender/swollen joint count will be analysed via a Poisson model fit via generalised estimating equations with an exchangeable working correlation structure to account for multiple measurements per participant. This model will include the baseline measure and the stratifying variables of site and gender, and include categorical terms for time, a term for randomised group and terms for the interaction between randomised group and time. If more than 5% of participants are missing their primary outcome, multiple imputation will be applied to account for missing data, with missing values imputed separately by treatment group. Sensitivity analyses will fit outcome regression models additionally adjusted for age, body mass index, and severity of radiographic OA and synovitis if baseline imbalances between randomised groups with respect to these variables are considered clinically important. A sensitivity analysis of the primary outcome will be undertaken to estimate the effect of methotrexate under the assumption of hypothetical complete compliance to treatment by applying a two-stage least squares instrumental variables approach [45], where full compliance will be defined as participants taking at least 80% of their medication.

### Data integrity and management

Data will be collected using prespecified case report forms. All data collected will be kept strictly confidential. Paper-based data will be stored in locked cabinets with secured and restricted access at each study site. Electronic data will be stored in a password-protected database with secured and restricted access. Participants will be identified by study ID and randomisation code, with other information with the potential of identifying individuals removed. Data transfer will be encrypted with all data de-identified. After trial completion, case report forms will be securely archived, and electronic data will be saved on the secure server, password-protected and accessible only to study investigators. There are no planned interim analyses and stopping guidelines.

### Dissemination

Trial results, regardless of statistical significance, will be published in peer-reviewed journals and presented at national and international conferences. Upon publication of the primary manuscript, participants will be informed of their group allocation and provided with the results.

## Discussion

This randomised controlled trial is conducted to determine whether 20 mg methotrexate weekly reduces pain and slows structural progression in participants with symptomatic hand OA and synovitis, the common inflammatory phenotype of hand OA.

Histological changes in hand OA with synovitis are very similar to those seen in rheumatoid arthritis [22], where methotrexate is first line disease-modifying anti-rheumatic therapy for most patients with rheumatoid arthritis. Methotrexate is a folate analogue that inhibits dihydrofolate reductase, required for purine and pyrimidine synthesis and cell proliferation [23]. The mechanism of action of methotrexate is considered to be due to inducible apoptosis in synovium, making it an effective treatment for synovitis [46]. Synovial biopsy samples from inflammatory hand OA show intense proliferative synovitis, indistinguishable from rheumatoid arthritis [22]. Increased levels of pro-inflammatory cytokines (such as tumour necrosis factor α, interleukin-1β and interleukin-6), reduced levels of anti-inflammatory cytokines (such as interleukin-10 and interleukin-1RA), infiltration of mononuclear cells and adaptive immune cell responses have all been demonstrated within OA fluid and tissue [47–49]. These data suggest that modulating the inflammatory response may be effective as a treatment target for hand OA. Therefore, therapeutics historically used to treat inflammatory arthritis may benefit patients with hand OA and synovitis.

The results from previous clinical trials of tumour necrosis factor blockers provide proof of concept that targeting synovitis is effective in reducing pain and slowing structural progression of hand OA [20, 21]. There is evidence that methotrexate improves symptoms and reduces radiographic progression of rheumatoid arthritis [23], and reduces pain in randomised controlled trials of knee OA [24, 25]. Methotrexate has been in clinical practice for approximately 40 years. Rheumatologists and general practitioners have extensive experience using methotrexate. In clinical practice, the most common reason for methotrexate discontinuation in a 13-year study was gastrointestinal side effects (10.8%) [50]. Administration of folic acid reduces adverse events [31], while folinic acid rescue can be used to reverse methotrexate [31]. In a systematic review, 3% of people on methotrexate monotherapy experienced a serious side effect compared with 2% of placebo-controls [23]. Sixteen percent of people discontinued methotrexate within 52 weeks due to an adverse event, compared with 8% of placebo subjects [23].

This study will provide high-quality evidence to address whether methotrexate has an effect on reducing pain over 6 months in patients with hand OA and synovitis, a common phenotype of hand OA. The trial has major clinical and public health importance. If found to be beneficial, the study findings will inform international clinical practice guidelines for the management of hand OA. While a positive trial would help to inform clinical guidelines internationally, a negative trial would be highly topical and change current trends in clinical practice.

## Data Availability

Data sharing is not applicable to this article as no datasets were generated or analysed during the current study.

## Abbreviations

AE: Adverse event
AUSCAN: Australian Canadian Osteoarthritis Hand Index
CONSORT: Consolidated Standards of Reporting Trials
HAQ: Health Assessment Questionnaire
FIHOA: Functional Index for Hand Osteoarthritis
MHQ: Michigan Hand Outcomes Questionnaire
MRI: Magnetic resonance imaging
NSAIDs: Non-steroidal anti-inflammatory drugs
OA: Osteoarthritis
OARSI: Osteoarthritis Research Society International
OMERACT: Outcome Measures in Rheumatology
VAS: Visual analogue scale
WOMAC: Western Ontario and McMaster Universities Osteoarthritis Index
